# Associations between *APOE* Variants and Metabolic Traits and the Impact of Psychological Stress

**DOI:** 10.1371/journal.pone.0015745

**Published:** 2011-01-19

**Authors:** Sofia I. Iqbal Kring, John Barefoot, Beverly H. Brummett, Stephen H. Boyle, Ilene C. Siegler, Søren Toubro, Torben Hansen, Arne Astrup, Oluf Pedersen, Redford B. Williams, Thorkild I. A. Sørensen

**Affiliations:** 1 Institute of Preventive Medicine, Copenhagen, Denmark; 2 Center for Pharmacogenomics, University of Copenhagen, Copenhagen, Denmark; 3 Department of Psychiatry and Behavioral Sciences, Duke University Medical Center, Durham, North Carolina, United States of America; 4 Research Clinic of Nutrition, Hvidovre University Hospital, Hvidovre, Denmark; 5 Hagedorn Research Institute and Steno Diabetes Center, Gentofte, Denmark; 6 Faculty of Health Science, University of Southern Denmark, Odense, Denmark; 7 Department of Human Nutrition, Centre of Advanced Food Research, Faculty of Life Sciences, University of Copenhagen, Copenhagen, Denmark; 8 Institute of Biomedicine, Faculty of Health Science, University of Copenhagen, Copenhagen, Denmark; 9 Faculty of Health Science, University of Aarhus, Aarhus, Denmark; Rikagaku Kenkyūsho Brain Science Institute, Japan

## Abstract

**Objective:**

In a previous study, we observed that associations between *APOE* rs439401 and metabolic traits were moderated by chronic stress. Thus, in a population of stressed and non-stressed Danish men, we examined whether associations between *APOE* rs439401 and a panel of metabolic quantitative traits, all metabolic traits which may lead to T2D and CVD were moderated by psychological stress.

**Methods:**

Obese young men (n = 475, BMI≥31.0 kg/m^2^) and a randomly selected control group (n = 709) identified from a population of 141,800 men were re-examined in two surveys (S-46: mean age 46, S-49: mean age 49 years) where anthropometric and biochemical measures were available. Psychological stress factors were assessed by a self-administered 7-item questionnaire. Each item had the possible response categories “yes” and “no” and assessed familial problems and conflicts. Summing positive responses constituted a stress item score, which was then dichotomized into stressed and non-stressed. Logistic regression analysis, applying a recessive genetic model, was used to assess odds ratios (OR) of the associations between *APOE* rs439401 genotypes and adverse levels of metabolic traits.

**Results:**

The *APOE* rs439401 TT-genotype associated positively with BMI (OR = 1.09 [1.01; 1.17]), waist circumference (OR = 1.09 [1.02; 1.17]) in stressed men at S-46. Positive associations were observed for fasting plasma glucose (OR = 1.42 [1.07; 1.87]), serum triglycerides (OR = 1.41 [1.05; 1.91]) and with fasting plasma insulin (OR = 1.48 [1.05; 2.08]) in stressed men at S-49. Rs439401 TT-genotype also associated positively with surrogate measures of insulin resistance (HOMA-IR; OR = 1.21 [1.03; 1.41]) and inversely with insulin sensitivity (Stumvoll index; OR = 0.90 [0.82; 0.99], BIGTT-S_I_; OR = 0.60 [0.43; 0.85]) in stressed men. No significant associations were observed in non-stressed men, albeit the estimates showed similar but weaker trends as in stressed men.

**Conclusion:**

The present results suggest that the *APOE* rs439401 TT-genotype is associated with an adverse metabolic profile in a population of psychologically stressed Danish men.

## Introduction

Obesity is associated with a number of deleterious consequences, such as development of metabolic diseases and type 2 diabetes (T2D), which may lead to cardiovascular disease (CVD) [1]–[3]. Abdominal obesity, insulin resistance and an adverse plasma lipid profile make up a cluster of metabolic traits that has been associated with increased CVD risk [3], [4]. *APOE* (chromosome 19q12-13.2) is known to have a key role in determining inter-individual differences in lipid metabolism [5], [6], and *APOE* gene variants may alter glucose metabolism [6], [7] moderated by obesity [8]–[10]. *APOE* gene variants may thus, be important in association studies of metabolic traits.

In a previous study, associations between *APOE* rs439401 TT-genotype and an adverse metabolic profile (increased waist circumference, triglycerides and insulin; decreased HDL-cholesterol) were observed in a sample of chronically stressed U.S. caregivers [11]. In order to elucidate the relationship between the *APOE* polymorphism and stress-related vulnerability to develop a detrimental metabolic profile further research will be required to replicate our previously reported associations. Thus, in the present study, we examined *APOE* rs769450, rs405509 and rs439401 in relation to obesity and related metabolic quantitative traits in a group of middle-aged Danish men according to their perceived stress levels.

## Material and Methods

### Study population

Among 362,200 Caucasian men examined at the mean age of 20 years at the draft boards in Copenhagen and its surroundings during 1943–77, a randomly selected group of one in every hundred men (n = 3,601) and all obese men (n = 1,930) were identified. Obesity was defined as 35% overweight relative to a local standard in use at the time, and this corresponds to a BMI≥31.0 kg/m^2^, which proved to be above the 99th percentile. All obese and half of the random sample, still living in the region, were invited to a follow-up survey in 1992–94 at the mean age of 46 years (survey S-46) and in 1998–2000 at the mean age of 49 years (survey S-49). The criteria for invitation to the follow-up surveys and the participation have been described previously [12]–[14], and the number of participants shows the expected attrition over time **(** **Table 1** **)**. Phenotypic assessments were carried out at all surveys, though most extensively at survey S-49. DNA was sampled from blood sample buffy coats at S-46. In total, 1,184 (475 obese and 709 randomly selected) participants were genotyped, indicating that the randomly selected group represent 141,800 men originally identified at the draft board examination. Among these 385 (143 obese and 242 randomly selected) had been assessed in S-49.

**Table 1:** Genotype distribution of *APOE* rs769450, rs405509 and rs439401 for controls and obese participants in absolute numbers and percentages at survey S-46 (n = 1,186) and S-49 (n = 385).

dbSNP	Alleles*	Location**	Controls				Obese			
**Rs769450**	A/G	17678662	*GG (%)*	*GA (%)*	*AA (%)*	MAF	*GG (%)*	*GA (%)*	*AA (%)*	MAF
S-46			251 (35.9)	323 (46.1)	126 (18.0)	0.41	157 (33.1)	232 (48.8)	85 (17.9)	0.42
S-49			86 (36.4)	97 (41.1)	53 (22.5)	0.43	48 (34.5)	66 (47.5)	25 (18.0)	0.42
**Rs405509**	G/T	17677054	*GG (%)*	*GT (%)*	*TT (%)*		*GG (%)*	*GT (%)*	*TT (%)*	
S-46			200 (28.3)	355 (50.3)	151 (21.4)	0.47	139 (29.1)	240 (50.2)	99 (20.7)	0.46
S-49			78 (32.2)	110 (45.5)	54 (22.3)	0.45	49 (34.2)	62 (43.4)	32 (22.4)	0.44
**Rs439401**	C/T	17682669	*CC (%)*	*CT (%)*	*TT (%)*		*CC (%)*	*CT (%)*	*TT (%)*	
S-46			313 (44.1)	324 (45.7)	72 (10.2)	0.33	195 (41.0)	215 (45.3)	65 (13.7)	0.36
S-49			107 (44.2)	107 (44.2)	28 (11.6)	0.34	64 (45.4)	55 (39.0)	22 (15.6)	0.35

*The minor alleles are shown in bold-faced letters.

**SNP position based on the Human Genome Build.

SNPs = single nucleotide polymorphisms, MAF = Minor allele frequency.

S-46 and S-49 denote the separate surveys in which participants were examined at the mean age of 46 and 49 years, respectively.

### Ethics Statement

The Danish Data Protection Agency and the Ethical Committees of Copenhagen and Frederiksberg municipalities approved the study, which was in accordance with the Helsinki Declaration II. All participants signed written consent before participating.

### Molecular genetic analyses

Genotyping of the *APOE* tagging SNPs (rs769450, rs405509, rs439401) were performed using Taqman allelic discrimination (KBioScience, Herts, UK). Genotyping was successful for rs769450, rs405509 and rs439401 on 1,184 participants. All genotype groups obeyed Hardy-Weinberg equilibrium (*P*>0.05; **Table 1**).

### Phenotypic measurements

Waist circumference (cm) was measured according to the WHO recommendations to the nearest 0.5 cm with the subjects standing, using a nonexpendable linen tape measure [15]. Participants in S-46 had non-fasting glucose and lipid levels determined on fresh plasma samples. In the S-49 cohort, an oral glucose tolerance test (OGTT) was conducted, except in individuals with diagnosed and therefore likely treated diabetes (n = 10) [12]. We also computed HOMA-IR and derived indices of insulin sensitivity according to Stumvoll [16] and the recently recommended BIGTT index [17]. Insulin sensitivity and acute insulin response were assessed by the recently recommended BIGTT indices (BIGTT-S_I_ and BIGTT-AIR, respectively) on the basis of measurements of plasma glucose and insulin at the time points 0, 30 and 120 minutes during the OGTT [17]. Details on data collections and measurement of anthropometric and biochemical variables have been described elsewhere [12], [18], [19].

### Psychological stress

Stress factors were assessed by a self-administered 7-item questionnaire **(** **Table 2** **)** checked with the participant by trained staff, and by various laboratory tests. Each item of the questionnaire that had the possible response categories “yes” and “no” assessed familial problems and conflicts (items given in table 2). Summing positive responses constituted a stress item score (range: 0–7), which was then grouped into two categories: 0 (n = 393) and ≥1 (n = 791) items positive in the present study. The stress variable was validated in the entire cohort of Copenhagen City Heart Study and was highly correlated with vital exhaustion, a psychological measure characterized by fatigue and depressive symptoms, that in a previous study has been associated with ischemic heart disease and all-cause mortality [20].

**Table 2:** The seven items in construct of stress in order of increasing prevalence.

“Do you have long-term…”	Prevalence (%)
Conflicts with children	4.4
Problems with children	6.4
Illness of children	11.4
Economic problems	16.7
Illness of yourself	19.0
Marital problems	21.9
Illness of family member	46.3

### Statistics

A likelihood ratio test for an additive, a dominant and a recessive effect of the genotyped variants determined that a recessive genetic model was chosen for rs439401, rs405509 and rs769450 (wild type and heterozygous genotype versus homozygous genotype).

In order to properly take into account the sampling design, the two groups of obese and controls have been analysed together for the logistic regression analyses, but separately for each follow-up survey S-46 and S-49. The massive enrichment of the right tail of the BMI distribution implies that the data cannot be analysed with BMI or BMI-associated outcomes as response variables in common regression models. Also, using a dichotomized case-control approach would waste considerable statistical efficiency otherwise gained by using the quantitative phenotypes. Hence, to take advantage of the greater statistical power and much wider coverage of the phenotypes, we reversed the statistical models for the associations and examined the probability of carrying the particular risk-allele genotype for a given level of the phenotypes. This can be done without distributional assumptions about the phenotypes. Thus, logistic regression analysis was used to assess the ORs of the genotype (response variable) in relation to the phenotypes (covariates) in the combined obese and randomly selected control groups. There were no indications of multicollinearity among the phenotypes. The purpose of the regression analyses was to estimate and test the specific hypotheses while controlling for relevant covariates. All analyses were stratified according to stress levels (stressed/non-stressed) and adjusted for age as a continuous metabolic variable. P-values<0.05 were considered statistically significant. Analyses were performed using SAS statistical procedures (version 9.1; SAS Institute Inc, Cary, NC).

## Results

### Descriptive characteristics

In **table 3** mean values of age, and metabolic traits are given for the pooled group of participants, and also separately for stressed and non-stressed participants for each survey. Only age at S-46 was significantly lower in stressed participants compared with non-stressed participants (p = 0.001).

**Table 3:** Study population characteristic given as mean±SD by stress status at survey 46 (S-46) and survey 49 (S-49).

	S-46			S-49		
	Pooled	Stressed	Non-stressed	Pooled	Stressed	Non-stressed
	*N = 1,184*	*N = 791*	*N = 393*	*N = 383*	*N = 258*	*N = 125*
Age (yrs)	47.1±8.1	46.8±7.6	47.8±9.0	49.3±5.8	49.5±5.9	49.0±5.7
**Metabolic traits**						
BMI (kg/m^2^)	29.9±6.2	30.0±6.4	29.6±5.8	29.8±6.6	29.6±6.7	30.1±6.2
Waist circumference (cm)	102.9±16.2	103.1±16.7	102.4±15.0	102.9±16.7	102.5±17.0	103.9±16.2
Plasma glucose (mmol/L)	6.3±2.6	6.4±2.7	6.2±2.3	5.9±1.1	5.9±1.2	6.0±0.9
Serum insulin (pmol/L)	-	-	-	49.8±39.7	49.5±42.2	50.5±34.2
Serum HDL cholesterol (mmol/L)	1.3±0.4	1.3±0.4	1.3±0.4	1.2±0.3	1.2±0.3	1.2±0.3
Serum triglycerides (mmol/L)	-	-	-	1.6±1.1	1.5±1.0	1.7±1.4
Systolic BP (mmHg)	142.7±18.4	142.5±18.0	143.3±19.1	126.6±17.8	125.7±17.5	128.5±18.4
**Derived indices**						
HOMA-IR	-	-	-	-	2.2±2.1	2.3±1.8
BIGTT-AIR	-	-	-	-	7.5±0.6	7.6±0.6
Matsuda index	-	-	-	-	6.7±4.6	5.9±3.3
Stumvoll index	-	-	-	-	7.0±3.3	7.1±2.8
BIGTT-S_I_	-	-	-	-	1.7±0.9	1.7±0.7

P-value for trend was >0.05 for all variables, except for age at S-46.

### Regression analyses for metabolic traits

Results from logistic regression analyses for metabolic traits are given as OR with 95% confidence intervals. Significant associations between participants homozygous for the minor T-allele for rs439401 were observed in stressed individuals for BMI and waist circumference in S-46 and fasting plasma glucose, insulin, triglycerides in S-49 **(** **Table 4** **).**

**Table 4:** Metabolic traits and OGTT-derived indices in relation to *APOE rs439401* at survey 46 (S-46) and survey 49 (S-49). OR (95% confidence intervals) in stressed individuals homozygous for the minor T-allele.

Variables	S-46StressedN = 791		Non-stressedN = 393		S-49StressedN = 258		Non-stressedN = 125	
	OR (95% CI)	*p*	OR (95% CI)	*p*	OR (95% CI)	*p*	OR (95% CI)	*p*
**Metabolic traits**								
BMI (kg/m^2^) *	1.09 [1.01; 1.17]	0.02	1.09 [0.98; 1.22]	0.12	1.05 [0.96; 1.16]	0.30	1.14 [0.94; 1.38]	0.18
Waist (cm) **	1.09 [1.02; 1.17]	0.01	1.10 [0.99; 1.23]	0.08	1.07 [0.97; 1.18]	0.16	1.15 [0.96; 1.37]	0.14
Plasma glucose (mmol/L)	0.98 [0.89; 1.08]	0.68	0.99 [0.97; 1.01]	0.33	1.42 [1.07; 1.87]	0.01	1.09 [0.60; 1.97]	0.77
Serum insulin (50 pmol/L)	-	-	-	-	1.48 [1.05; 2.08]	0.03	1.39 [0.63; 3.06]	0.42
Serum HDL cholesterol (mmol/L)	0.71 [0.38; 1.33]	0.29	0.56 [0.22; 1.41]	0.22	0.37 [0.09; 1.49]	0.16	0.47 [0.07; 3.09]	0.43
Serum Triglycerides (mmol/L)	-	-	-	-	1.41 [1.05; 1.91]	0.02	0.87 [0.46; 1.66]	0.67
Systolic BP (10 mmHg)	1.00 [0.98; 1.01]	0.94	1.10 [0.92; 1.30]	0.30	1.02 [0.83; 1.24]	0.88	1.27 [0.95; 1.69]	0.11
**Derived indices**								
HOMA IR	-	-	-	-	1.21 [1.03; 1.41]	0.01	1.13 [0.83; 1.54]	0.43
BIGTT-AIR	-	-	-	-	0.81 [0.47; 1.38]	0.44	0.97 [0.34; 2.77]	0.95
Stumvoll index (IS)	-	-	-	-	0.90 [0.82; 0.99]	0.02	0.89 [0.73; 1.10]	0.28
Matsuda index (IS)	-	-	-	-	0.91 [0.83; 1.01]	0.08	0.90 [0.73; 1.11]	0.31
BIGTT-S_I_	-	-	-	-	0.60 [0.43; 0.85]	0.004	0.69 [0.31; 1.52]	0.36

BMI = body mass index, BIGTT-AIR = OGTT-derived index of acute insulin response, BP = blood pressure, BIGTT-S_I_ = OGTT-derived index of insulin sensitivity, IS = insulin sensitivity.

*: Per two-unit increment of BMI,

**: Per five cm increment of waist circumference.

In S-46, a two-unit increase in BMI (kg/m^2^) was positively associated with increased odds of 9% for being homozygous for the minor T-allele in stressed individuals (OR = 1.09 [1.01; 1.17]; Table 4). Likewise, each five cm increment in waist circumference was positively associated with increased odds of 9% for being homozygous for the minor T-allele in stressed individuals (OR = 1.09 [1.02; 1.17]).

In S-49, one unit increase of glucose and triglycerides (mmol/L) was positively associated with increased odds of 42% and 41%, respectively, for being homozygous for the minor T-allele in stressed individuals (glucose: OR = 1.42 [1.07; 1.87]; triglycerides: OR = 1.41 [1.05; 1.91]. Further, one unit increase of insulin (50 pmol/L) was positively associated with increased odds of 48% for being homozygous for the minor T-allele in stressed individuals in S-49 (insulin: OR = 1.48 [1.05; 2.08]). HDL-cholesterol and systolic blood pressure were not significantly associated with rs439401 in either survey. No significant associations were observed among non-stressed individuals. No significant associations were observed for rs769450 or rs405509.

### Regression analyses for derived indices

Derived indices for insulin resistance and insulin sensitivity revealed significant results, in S-49. In stressed individuals, one unit increase in HOMA index for insulin resistance was positively associated with increased odds of 21% for being homozygous for the minor T-allele (OR = 1.21 [1.03; 1.41]). One unit increase in Stumvoll index for insulin sensitivity was inversely associated with decreased odds of 10% for being homozygous for the minor T-allele in stressed individuals (OR = 0.90 [0.82; 0.99]). Similar estimates were observed when insulin sensitivity was assessed as Matsuda index, albeit borderline significant, only. These results were strongly confirmed when using the OGTT-derived measure for insulin sensitivity (BIGTT-S_I_), where one unit increase in BIGTT-S_I_ was inversely associated with decreased odds of 40% for being homozygous for the minor risk T-allele in stressed individuals (OR = 0.60 [0.43; 0.85]). No notable associations were observed among non-stressed individuals.

## Discussion

The present study of Danish men confirmed associations of *APOE* rs439401 with quantitative metabolic traits [11], which were also observed in a recent European genome-wide association study of blood lipid levels [21]. The rs439401 TT-genotype was positively associated with obesity, assessed as BMI and waist circumference at survey S-46. Although estimates were almost similar, the results from S-49 did not reach statistical significance, with regard to BMI and waist circumference. Positive associations were also observed for the rs439401 TT-genotype in relation to fasting plasma glucose, insulin and triglyceride levels at survey S-49. All associations were, however, only present in stressed Danish men compared with non-stressed men. Further, associations of the TT genotype with surrogate measures of whole body insulin resistance and insulin sensitivity were observed only in stressed participants.

The present results are in agreement with our first study of *APOE* rs439401 and metabolic traits of T2D and CVD [11]. In our previous study, the rs439401 TT-genotype was associated with elevated waist circumference, insulin, and triglyceride levels and decreased HDL-cholesterol level among chronic stressed men and women compared with carriers of the rs439401 C-allele. It is not clear in the present data of stressed Danish men why associations of the TT-genotype with BMI, glucose and HOMA-IR were not found in stressed U.S. caregivers.

One possible contributor to the different patterns of associations is the nature of the psychosocial stressors in the two study populations. Being the primary caregiver for a relative with Alzheimer's disease or other major dementia is a life stressor that is chronic, unremitting and pervasive for the caregiver [22]–[24]. In contrast, while the stressors used to define stress versus no stress in the Danish sample have effects on health, it is likely that they are less severe and pervasive in daily life than being the caregiver. It is also possible that, in addition to the metabolic consequences of obesity, it was only in men with high general psychosocial stress and the added particular stress imposed by being obese that the effects of rs439401 genotype were present. Overall, the present results in Danish men accomplish our goal of replication by showing a similar pattern of association between *APOE* rs439401 and metabolic traits that is moderated by stress.

The strengths of the present population-based study of Danish men include the repetitive detailed assessment of anthropometric and physiological variables at mean ages 46 and 49 years for the same individuals in different subsets of the cohort, which makes the present study population unique and appropriate for investigating the impact of gene variants on body weight and related metabolic traits. Several limitations, however, need to be acknowledged. The S-46 includes a much less demanding examination program and the participants cover a much broader part of the original cohort, whereas the S-49 was a very intensive and demanding examination program, including for example ventilated hood measurements of metabolic rates and VO_2_ max measurement, for which we needed to select participants capable of completing such program [12]. This selection process may lead to differences in the phenotypic measurements.

Although the hypotheses of our study were directly derived form the previously published study of chronically stressed American care-givers [11], the finding may still be considered exploratory and therefore need further replication. Population stratification may occur due to systematic admixture of ancestry and lead to spurious associations [25]. However, population stratification is unlikely to explain the obtained results due to the homogenous study population of Danish Caucasian men, in which the examined genotype distributions complied with Hardy-Weinberg equilibrium. The present sample size may seem small for a genetic association study, but was able to confirm the association between rs439401 TT genotype and an adverse profile of metabolic traits only in stressed persons found in our previous study [11]. Keeping the phenotypes as quantitative variables in the analyses, however, the efficiency is considerably higher as reflected in the fairly narrow confidence intervals, which means that we thereby have narrowed down the likely true ORs that could have given rise to the observed ORs. The metabolic traits associated with obesity may be inter-correlated to various extents, but according to a recent twin study [26], there is little common underlying genetic or shared environmental etiology behind these correlations, which we think justifies the separate analysis of each of the traits as we have conducted.

In conclusion, the results from the present study confirmed that *APOE* rs439401 associated with a panel of metabolic traits, moderated by psychological stress. The results were a replication of our previous study of chronic stressed U.S. caregivers in whom a similar adverse metabolic profile was observed. If confirmed in further research, particularly in a prospective study with sufficient incidence of T2D and CVD over time, the findings in these two studies suggest that the *APOE* rs439401 TT-genotype might be used to identify persons at high risk of developing T2D and/or CVD who might be targeted for preventive interventions.
